# Helpless infants are learning a foundation model

**DOI:** 10.1016/j.tics.2024.05.001

**Published:** 2024-08

**Authors:** Rhodri Cusack, Marc’Aurelio Ranzato, Christine J. Charvet

**Affiliations:** 1 Trinity College Dublin, Dublin, Ireland; 2 Google DeepMind, London, UK; 3 Auburn University, Auburn, AL, USA

**Keywords:** infant, evolutionary biology, development, neuroimaging, machine learning, pre-training

## Abstract

Humans have a protracted postnatal helplessness period, typically attributed to human-specific maternal constraints causing an early birth when the brain is highly immature. By aligning neurodevelopmental events across species, however, it has been found that humans are not born with especially immature brains compared with animal species with a shorter helpless period. Consistent with this, the rapidly growing field of infant neuroimaging has found that brain connectivity and functional activation at birth share many similarities with the mature brain. Inspired by machine learning, where deep neural networks also benefit from a ‘helpless period’ of pre-training, we propose that human infants are learning a foundation model: a set of fundamental representations that underpin later cognition with high performance and rapid generalisation.

## Highlights

Human infants are helpless for a protracted period after birth. This has been attributed to maternal constraints causing an early birth while the brain is still immature.
However, anatomical studies have shown that relative to other species, human newborn brains are not particularly immature.
Consistent with this, neuroimaging studies have shown that the structure and function of many cognitive systems is surprisingly mature in humans at birth.
Why else might humans have a protracted helpless period? In machine learning, learning good representations of the environment through self-supervised learning in large deep neural networks yields enormously powerful ‘foundation models’, which allow better generalisation to new problems and have a higher final performance.
We propose that in the helpless period, human infants are learning a foundation model that underpins their later cognition.

## Why are human infants helpless for so long?

**Figure 1:**
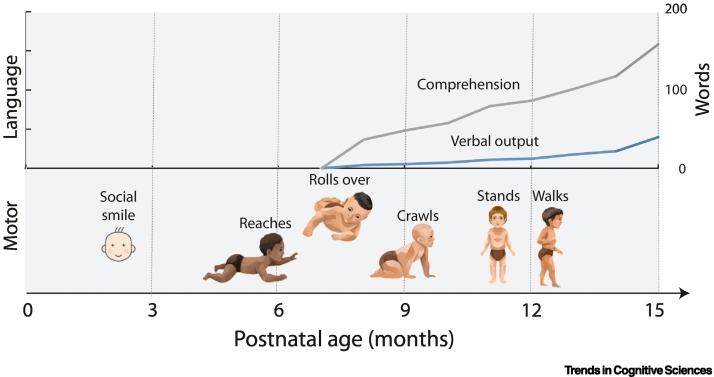
Many cognitive functions are slow to develop in humans. Language and gross motor behaviours such as crawling and walking emerge in the second half of the first year [1,3].

Humans have a protracted period of **helplessness** (see Glossary) during infancy: while chickens and horses can walk on the day they are born, humans do not typically roll over until 7 months old or walk until 1 year old [1]. Despite hearing hundreds of thousands of words per month [2] and the enormous value that a few words could confer, language develops slowly over multiple years (Figure 1) [3]. Infants are limited in working memory and other executive functions [4]. This limited repertoire of **adaptive behaviour** leaves them helpless, putting them at risk and placing a burden on their caregivers. Despite this, throughout the course of human evolution, this protracted period of reliance has survived **behavioural extinction**. We review longstanding explanations for human helplessness and highlight their weaknesses. We then review recent converging evidence from evolutionary biology, neuroimaging of infants, and machine learning, which points towards a different driver for helplessness: the need to develop foundational mental representations.

## Brain development in humans and other species

**Figure 2:**
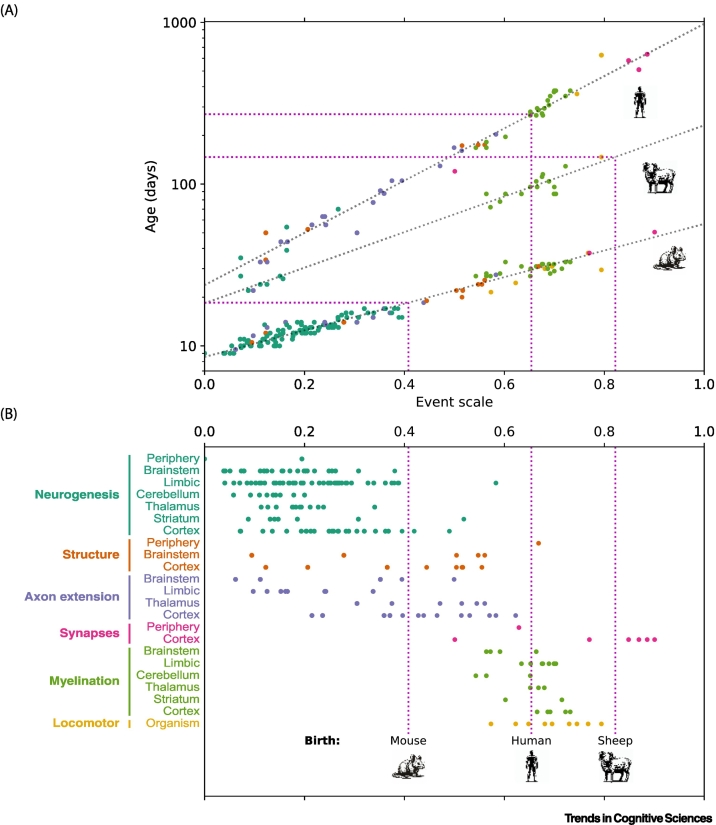
Human newborns have moderately mature brains but further development occurs postnatally. (A) A model compared the timing of 271 neurodevelopmental events across 18 mammalian species [8,87]. The ‘event scale’ quantified the order and relative timing of neurodevelopmental events across species and was found to be strongly predictive of event timing in each species, capturing a large proportion of the variance. The human, sheep, and mouse are shown as examples. The intercept and slope of the line relating the event scale to the log-transformed age of events (expressed in days post-conception) varies across species. Points directly vertically above each other (i.e., at the same event scale value) corresponded to the same neurodevelopmental event, although some are missing as not all neurodevelopmental events were measured in all species. In contrast to this broad alignment of the timing of events across species, birth was heterochronous and happened at distinctly different points in neurodevelopment (see pink dotted lines). (B) These neurodevelopmental events capture many but not all developmental processes and are categorised by class and brain structure. By the time of birth in humans (central pink dotted line), most structures have undergone neurogenesis and axon extension. There is considerable synaptogenesis and myelination in the first postnatal year in humans. Other species such as mice are born relatively earlier than humans and have yet to undergo axon extension followed by myelination. Some structures (e.g., cerebellum, hippocampus) undergo neurogenesis for an extended duration, and the time points shown here focus on the timetable of embryonic neurogenesis (i.e., when most of the neurogenesis occurs).

Helplessness in humans has long been attributed to maternal constraints. The obstetrical dilemma theory proposes that because efficient bipedal walking limits the size of the pelvis and thus the birth canal, and because humans have big heads, babies must be born early while their brains are still immature [5]. A theory focussing on another maternal limit, uterine energy delivery, also argues for early birth, leading to brain immaturity in infancy [6]. According to both theories, human infants are helpless for a protracted period because their brains are immature. They posit that humans are exceptional at their time of birth, but this is not supported by comparisons of brain development across species, which instead place humans on a continuum [7]. Although the overall pace of development varies widely across species, the order and relative timing are similar for a broad range of neurodevelopmental events, including neurogenesis, axon extension, myelination, synaptogenesis, brain growth, and locomotion (Figure 2A). These events have therefore been aligned across species onto a common scale called the **event scale**. A model is fit to the age of neurodevelopmental events and the event scale. Predictions from this model correlate well with empirical times (*r* = 0.993) [8,9] (www.translatingtime.org).

Although most events align well, in some species specific biological and behavioural events occur unusually early or late. These so-called **heterochronies** account for cross-species variation in life history, brain structure, and behaviour. Birth is heterochronous in many species, varying relative to the timeline of other developmental events [10]. **Altricial** species such as rats and mice are born in an immature state with many key developmental processes occurring postnatally, whereas **precocial** animals such as sheep are born in a relatively mature state with key developmental processes occurring prenatally. Humans are relatively precocial in that human birth is actually relatively late compared with many other species. Indeed, key stages of brain development, including neurogenesis and axonal extension, are well advanced by the time humans are born (Figure 2B) [7,8], which suggests that species-specific effects, like obstetrical factors due to bipedal walking, are not dominant in accounting for infant helplessness. Yet, human newborns are helpless for many years.

Human newborns have been characterised as altricial, precocial, and secondarily altricial [7]. Such reported discrepancies in the state of maturity at birth may be accounted for by heterochronies in biological pathways. They are precocial in that their sensory systems are delivering rich streams of information by birth, but the human locomotor system is immature, with many milestones emerging after birth (Figure 1, Figure 2). Accordingly, humans have a relatively extended duration of helplessness in which their sensory systems are delivering rich streams of information from which to build foundational mental representations. A limitation of comparative work, however, is that it is restricted to neural markers that are compared across species. It can therefore be complemented by measures that may be specific to humans and are more specific with regards to cognitive systems (e.g., language networks). These are available from developmental neuroimaging.

## Measuring the emergence of cognition with neuroimaging

### Structural connectome

To assess white matter maturity in humans in more detail, we turn to infant neuroimaging, which has rapidly advanced in recent years. The structural connectome of the neonatal brain, as measured with diffusion-weighted MRI, has been found to be substantially similar to the adult’s [11]. Even subtle differences in the connectivity of specific local brain regions seen in adults [12] are present in infants [13]. However, considerable myelination is required, which progresses rapidly across the brain from superior to inferior and from posterior to anterior in the year after birth, with association cortical areas maturing for a relatively extended duration [14]. The neonates’ structural connectome is quite mature, but some aspects develop postnatally.

### Functional networks and activation

Given the reasonable maturity of white matter, how mature is brain activity in infants? Functional MRI (fMRI) in adults has shown that a small set of **resting-state networks (RSNs)** that each comprise brain regions with synchronously fluctuating activity can account for a considerable proportion of the signal variance. In neonates, adult-like RSNs have been found in the auditory, visual, and motor systems [15,16] and higher cognitive functions like the executive control network [17,18]. Using graph-theoretic analyses, key characteristics of adult brain networks, such as their ‘rich-club’ structure, are already found in newborns [19]. Thus, although not completely mature [16,18], functional networks are present in the infant brain. But are infant cortical networks already processing information from the environment?

Stimulation-evoked brain responses can also be measured with fMRI. In the auditory cortex, in the first months, sounds evoke activity [20,21] and rich acoustic features are encoded [22]. In the somatomotor system, stimulating the skin was found to evoke activity in neonates [23], while stimulating different limbs revealed a somatotopic organisation, even in preterm infants [24]. Strong visual responses have been found, with retinotopic organisation in both the dorsal and ventral streams from as early as 5 months [25,26]. There is sensitivity to visual flow-motion by 2 months [27], rich responses to movies by 4–12 months [28], and selective responses to categories such as faces, places, and bodies by 2–9 months [29,30]. This widespread stimulus-evoked activity suggests representation learning in many regions. There is also evidence of cognition beyond sensory systems. In the memory system, visual sequence learning modulated hippocampal activity as young as 3 months [31]. In executive control regions, visual attention recruited the prefrontal cortex early in the first year [32]. In sum, in the first months, cortical systems are engaged in both sensory processing and higher cognition.

### Atypical development

In addition to studying normative development, it is valuable to consider atypical trajectories [33]. Patients from the neonatal intensive care unit are at higher risk of brain injury and at higher risk of cognitive impairments. The development of motor function is slowed by a disruption to functional connectivity at birth [34,35], in either sensorimotor or executive control networks, suggesting that both support motor learning in the first months [34].

Across these diverse neuroimaging measures, a consistent picture is emerging that the brain is not too immature to function. To the contrary, even higher-order cognitive systems are already processing stimuli. This supports another view, aligned with the perspective of many developmental psychologists, that helpless infants are actively engaged in learning. But how are they learning and why do they remain helpless?

## The emergence of foundation models

Computational modelling of the brain has increasingly exploited **deep neural networks (DNNs)** engineered for practical applications in machine learning [36., 37., 38., 39., 40., 41., 42.]. These can achieve human-like performance on specific tasks and remarkably, with no modification, have proven effective models of brain activity in some domains, using simple linear mappings between the representations in DNNs and the brain [41,43].

**Figure 3:**
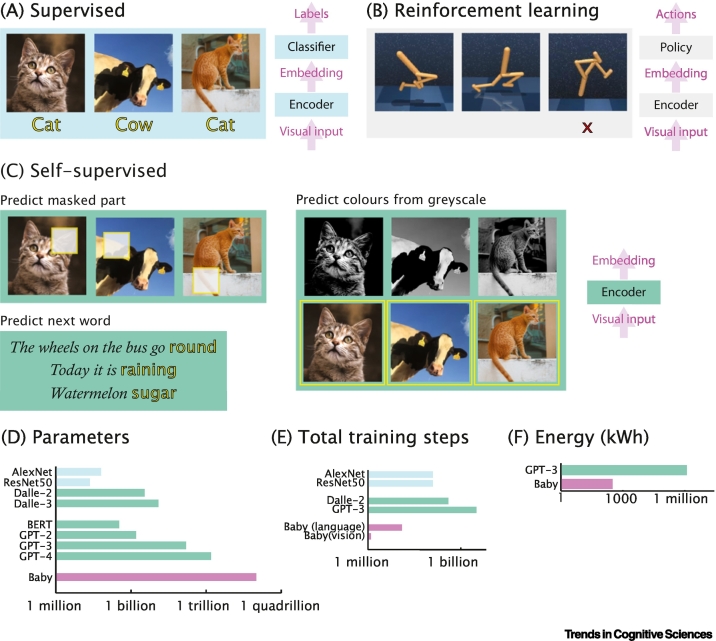
A comparison of machine self-supervised learning strategies. (A) Supervised learning requires each image to be associated with a label, which the model is trained to predict. (B) In reinforcement learning, the agent takes actions in the environment and its observations depend on the sequence of actions that it has taken. Learning proceeds by maximising the reward provided by the environment. (C) There are many self-supervised learning strategies, for learning from data streams themselves. (D) Self-supervised models benefit from a large number of parameters, but even the largest models do not reach the number of synapses in the infant brain (which roughly conceptually map onto the number of weight parameters in the artificial neural network) [50,60,117., 118., 119.] (https://openai.com/research/dall-e). (E) Self-supervised models require vast training datasets with many repetitions of each example leading to many more training steps [48,54,58,117,119]. Baby estimates are that one new object is seen every 10 s for 10 waking hours per day; and 1 million words are heard each month. (F) They also consume thousands of times more energy [119] than a baby in its first year; 100 calories/kg/day [120] and a UK boy’s growth trajectory (https://www.rcpch.ac.uk/sites/default/files/Boys_0-4_years_growth_chart.pdf), 270 000 calories in first year. Blue bars: supervised; green: self-supervised; and pink: baby.

In machine learning, three strategies for training DNNs are often differentiated: **supervised learning** (i.e., mapping inputs to labels); **reinforcement learning** (i.e., acting in the environment to maximise reward); and **self-supervised learning** (i.e., learning patterns within the input itself) (Figure 3) [44]. For all learning strategies, the networks initially process their input in an **encoder**, which derives a lower dimensional **embedding** that encodes the most relevant features of the input. Early progress was dominated by supervised training [45], but recently self-supervised training has grown in prominence and it has been called the ‘dark matter of intelligence’ [46].

For example, visual object recognition of the kind important for self-driving cars was long dominated by supervised learning, with datasets like ImageNet [47], where each image is associated with a single label [48,49]. More recently there has been a shift to self-supervised learning from larger datasets that do not have labels [50., 51., 52., 53.]. Generative diffusion models have learned how to create images from text prompts, such as DALLE [54] or MidJourney (https://www.midjourney.com/).

Self-supervised learning has led to tremendous advances in natural language processing [55,56], with tools such as the GPT family (https://cdn.openai.com/research-covers/language-unsupervised/language_understanding_paper.pdf) spawning an industry of new applications. Training these networks takes a considerable amount of time, computing resources [57], and huge quantities of (unlabelled) data, but once learned their representations are highly adaptable and have been used as the basis for improved performance on many supervised [58] and reinforcement learning tasks [59].

The process of training on large volumes of data by predicting the input itself prior to further adaptation to the task of interest is known as ‘**pre-training**’, and large unsupervised pre-trained models have become known as ‘**foundation models**’ [60]. Foundation models bridge a longstanding gap between humans, who can learn a new task with just a few examples, and machines, which until now had needed thousands of labelled examples [61].

## Self-supervised infant learning

Infants typically have an extremely limited vocabulary until 8 months old [3,62], precluding supervised learning with word labels before this age (Figure 1). They have a very limited behavioural repertoire, limiting the potential for reinforcement learning from gross motor actions (Figure 1) [1]. However, rich streams of information are available from the senses: prenatally for sounds [63]; after birth for vision, (with rapid development in acuity and colour sensitivity in the month after birth) [64,65], touch [23], and taste [66]. This provides a rich opportunity for self-supervised learning.

We propose that in the helpless period, infants are learning their own foundation models, developing the representations that will facilitate the subsequent rapid development of complex tasks such as walking and talking. As in machines, the benefit will be better and more efficient generalisation to tasks learned later in life. This proposal aligns with cognitive theories that emphasise the value of identifying patterns in perceptual inputs as a foundation for concept learning [67., 68., 69., 70., 71.].

We propose that helplessness is intimately connected to the use of self-supervised learning. Initial learning is like that in the encoder block in a self-supervised DNN (Figure 3C, right). However, the representations this develops are not yet connected to outputs and are therefore not acted upon. For example, they are not mapped onto discrete categories, as in the classifier block of a supervised DNN (Figure 3A, right), or used to select actions, as in the policy block of a deep reinforcement learning network (Figure 3B, right). Self-supervised learning is protracted and cognitive functions such as language recognition and motor control do not begin to emerge until its completion. The presence of distinct stages mirrors the surprising discovery from DNNs, of the degree to which sophisticated knowledge acquisition can result from the combination of passive observations and a simple initial learning rule, which prepare the system well for a diverse range of subsequent tasks.

### Predictions

A prediction from this two-stage theory is that additional brain systems should be recruited after the helpless period to implement supervised learning. Candidate systems are the anterior or medial temporal lobes for classification [72] and the frontal lobes for action policies [73]. Another way to test the theory will be to compare representation learning in DNNs and the brain. Representational similarity analysis [74] has been used to investigate whether supervised or self-supervised learning yields a better model of adult visual representations [36,75]. This strategy could be extended to infant learning, by measuring developing representations earlier in the lifespan, using neuroimaging [36,76] or eye movements [77], to test the two-stage prediction of early self-supervised and later supervised learning. At present these models are at an abstract level and not biologically plausible, but future computational models have the potential for an even deeper parallel at the implementational level, given the active investigation of biologically plausible mechanisms for backpropagation (used to update weights in DNNs) [78] and self-supervised learning [78., 79., 80.].

In machine learning, larger DNNs benefit more from self-supervised pre-training [81,82] (Figure 3D). This is intuitive, as complex networks can solve any given task in a wide variety of ways and often overfit to the idiosyncrasies of a particular dataset, when trained directly in supervised mode. Once a network overfits, it will poorly generalise to new instances of the same task, but it will also poorly adapt to subsequent tasks. Developing a generic, rich, and task agnostic representation of the environment, using self-supervised pre-training on large quantities of data, enables the network to rapidly adapt to a large variety of downstream tasks. To be effective, the distribution of the training data for the self-supervised learning should be sufficiently broad to include the type of data on which the downstream tasks will be performed, providing a further driver for an extended period of experience.

We hypothesise that diverse cognitive functions will benefit from initial self-supervised learning. For example, self-supervised models are now the best machine learning models for visual recognition [50., 51., 52., 53.]. In infants, vision provides a rich stream of information from birth. Although acuity is initially low, it has been proposed that this is adaptive in that it biases learning towards low-spatial frequency features valuable for key downstream tasks such as face recognition [83]. Furthermore, acuity improves rapidly over the first months, which could facilitate wider visual learning. A challenge to this idea is that visual representations appear to be quite similar in animals with a shorter helpless period, such as the macaque [75]. Future work could examine similarities and differences across species, with the prediction that representations in species with a longer helpless period will contain features that underpin performance in human-specific visual skills.

A second example is motor function. We propose a benefit to motor control based on evidence from machine learning. Deep reinforcement learning can be used to train simulated agents for motor tasks like walking or running (Figure 3B) [84]. If a DNN is taught to do one of these tasks, it does not generalise and requires complete retraining to do another one [85]. To create an agent that can easily adapt to many different tasks, a solution is to initially train the DNN in a self-supervised way by watching without acting and to only later fine-tune with action and rewards on the specific tasks [86]. This proof-of-principal shows that sometimes the best long-term performance, even in a motor task, is obtained by a protracted action-free helpless initial training phase. If similar constraints apply to infants, optimal learning may be a source of evolutionary pressure for a protracted initial helpless period. However, the effect of motor-specific delays in humans should be evaluated. Unlike arboreal primates, humans are bipedal and there is evidence that differences between species in locomotor capabilities are linked to heterochronies during development [10,11]. Moreover, human brains grow extensively during the first 2 years of life [9,87]. The relatively extended duration of some locomotor milestones and the extensive amount of brain growth in the first few years of life account for major evolutionary modifications in the human lineage, which include bipedal locomotion and human brain expansion. Future work might try to characterise the relative strength of general versus more specific evolutionary pressures.

A third example is general intelligence. Individual differences in how problems are represented are known to affect problem solving performance in intelligence tests [84]. Across species, a longer helpless period has been found to be associated with higher intelligence in adulthood [85]. A parsimonious combination of these ideas is that early self-supervised learning leading to better representations for general problem solving.

### Comparisons to other theories

The transition from self-supervised to supervised that we propose parallels Gopnik’s classic theory that posits childhood reflects a shift from exploration to exploitation, following the trajectory of the ‘simulated annealing’ optimisation algorithm [86,88]. A challenge with gradient-descent optimisation is that it is susceptible to finding solutions that are only locally optimal and worse than a more distant global optimum. Simulated annealing overcomes this by initially allowing large random steps ‘uphill’, away from the apparent optimum. By gradually reducing this randomness (‘annealing’), the algorithm can find more globally optimal solutions. Like our theory, simulated annealing predicts an initial lack of a drive towards convergence on goals will reap longer-term benefits, with an initial early phase of exploration of the optimisation landscape providing an investment that in the long run allows better learning. However, our theory and Gopnik’s simulated annealing differ in other predictions. Simulated annealing does not make predictions about the learning mechanism (i.e., self-supervised versus supervised) or predict qualitative change in learning mechanism at the end of the helpless period.

Another theory proposes that the driver for the extended helpless period in humans is social learning [89]. Like our proposal, this theory characterises the extended helpless period as adaptive and emphasises the shaping of the infant brain beyond its innate beginnings through learning. Our proposal differs in the importance attributed to social versus non-social knowledge. We believe that social learning is but one facet of learning and that a great deal of information is also available to the infant from non-social sources. We make the prediction that representations of important aspects of the non-social world will develop in infancy at least as quickly as social ones and that non-social brain systems will be just as activated in daily experience as social brain systems. We agree with [89] that identifying the effect of actions (contingency) and curiosity-driven learning will all be important, but suggest these extend to non-social learning too.

### Application to other species

Although this review has focussed on helplessness in human infants, other species likely also use self-supervised learning to develop representations that facilitate generalisation and enhance intelligence. For example, ravens are intelligent tool-using birds and they spend a considerable time (more than a month) in the nest being fed by both parents [90]. However, compared with humans, the time available in other species for self-supervised learning is limited, as even when the brain is quite mature at birth (e.g., sheep, Figure 2A), the absolute time taken to develop is still shorter: the slope that relates the time of neurodevelopmental events on the event scale to the natural logarithm of age in days is 2.91 for sheep, compared with 3.72 for humans [8]. Hence, postnatal neurodevelopmental events will be more compressed in time for these animals compared with humans. It would be helpful to determine the most accurate measure of when self-supervised learning stops and when task-driven fine-tuning begins. The timing and speed of this transition could be quantified by using neuroimaging and computational modelling to derive the balance of learning mechanisms by tracking changing brain representations. To generalise across species, it would be helpful to then identify a more accessible behavioural proxy. Quantifying the duration of helplessness across species and how it affects the quality of foundation models could reveal in turn how this affects intelligence and the extent to which it is a candidate for what makes humans special.

## Concluding remarks

Species with larger brains take longer to develop, but by the age of term birth in humans, neuroimaging has found that many structural and functional networks are present and cognitive systems are processing input from the senses. These observations suggest that during the helpless period, infants are learning in a self-supervised way from their environment. In parallel, in the field of machine learning, it has been recognised that foundation models (large DNNs pre-trained using self-supervised learning) can rapidly generalise to different tasks, which is pushing new frontiers in artificial intelligence. Foundation models amortise the large cost of pre-training by enabling very rapid and cheap adaptation to a wider spectrum of downstream tasks. In sum, converging evidence supports the view that human infants have a protracted helpless period so that they can learn a foundation model that underpins flexible, intelligent cognition later in life.

Many strategies for the training of self-supervised DNNs have been proposed (Figure 3 and Box 1). For language processing, predicting masked parts of a speech stream [91], the next word (https://cdn.openai.com/research-covers/language-unsupervised/language_understanding_paper.pdf), or the distribution of words around a given word [55]; for vision, predicting masked parts of an image from the remainder [53,92], predicting a frame of video from the previous ones [93], clustering on visual similarity [94], or predicting one colour channel from another [95]; and in cross modal datasets, predicting a caption from video [51]. One important general finding is that generic architectures (e.g., transformers) and objectives (e.g., prediction of what is coming next in a given input stream) can lead to broader downstream generalisation, if the neural network is large and if it is exposed to a sufficiently large set of observations. This provides a rich set of hypotheses on objective functions that infants could be using for self-supervised learning, which in the future can be tested by quantifying developing representations in DNNs and the infant brain and comparing them with representational similarity analysis [96]. It will build on the rapid expansion of computational cognitive neuroscience in adults, to open a new avenue in which to make conceptual and empirical advances in developmental science.

Box 1 **Self-supervised learning in machines and infants**

Self-supervised learning in DNNs uses a pretext task that is not important *per se*, but which leads to representations valuable for downstream tasks. Key classes are:

• Autoencoders, which encode the input into a lower-dimensional embedding and then decode to faithfully reconstruct the original. The embedding forms an information bottleneck, necessitating the compression of the input into a compact representation, given environmental statistics. Variants include masked autoencoders, in which part of the input is masked before encoding (Figure 3C, top left) [92] requiring the network to fill in missing parts; and sparse/variational autoencoders, which constrain the distribution of the embedding [110,111].
• Generative models, which generate text, images, or other content for a given context, using a decoder block. This might be a prediction. For example, the next word in a sentence, or an image, given a text prompt. With sufficiently large models and datasets, the representations learned are valuable for many downstream tasks.
• Contrastive learning, in which embeddings of pairs of related stimuli (e.g., the same object from different views [36,53] or modalities [51]) are made more similar, while unrelated pairs (e.g., different objects) are made more dissimilar.

A key consideration in self-supervised learning is what streams of information are being used. Multiple brain systems may use different streams, to independently develop domain-specific representations. Contrastive learning can capture visual representations [36,43] and multimodal vision-word learning [112]. Predictive coding, as used in generative models, has been proposed for many brain systems, including speech [113]. To understand self-supervised learning in infants, we will need to:

• Measure sensory input. New portable and laboratory-based recording tools are allowing for a richer characterisation of the multimodal streams infants learn from. Further, infants are not passive observers but shape their input through curiosity to optimally drive their learning [114].
• Measure brain responses to rich stimulus sets. This will allow for a more detailed characterisation of brain representations using methods such as representational similarity analysis [74], allowing learning architectures to be differentiated.
• Develop models. Existing models, engineered to solve practical problems using the current computer hardware, will not be perfect models of the brain.
• Understand how innate brain structure shapes learning, for example, as a ‘proto-organisation’ for sensory processing. Structure can be measured in newborns or fetuses using neuroimaging.
• Understand the role of replay [115] and dreaming [116]. This can be valuable in learning (e.g., by preventing new experiences overwriting existing knowledge).

Alt-text: Box 1

Despite the rapid pace of discovery in machine learning, it faces substantial challenges. Training foundation models in machines consumes enormous quantities of energy [57] and requires enormous quantities of data [97]. Human infants, in contrast, are much more efficient, needing tens of thousands of times less data and energy [2] (Figure 3E,F). Furthermore, foundation models sometimes fail catastrophically on tasks humans find easy. One characterisation is that they lack ‘common sense’ and a promising direction is to use infant learning as inspiration for more human-like learning in machines [98], beginning by finding benchmarks with which to compare humans and machines [99]. To address each of these challenges, it will be important to translate discoveries in developmental science to the field of machine learning [100]. This will require not just the measurement of the overt behaviour of infants from developmental psychology but also the measurement of quiescent changes in brain representations that do not yet manifest in behaviour, such as neuroimaging.

The rich streams of information from the senses provide considerable opportunity for self-supervised learning in infants. However, there are likely also contributions from social reward signals from interactions with caregivers. Young infants see faces for a substantial proportion of their early visual experience [101] and they are driven to seek social interaction from an early age [102]. Indeed, the benefit from extended social interaction with caregivers has been suggested as a driver for the length of the helpless period and increased social cooperation [103]. More generally, infants are not passive but are actively learning from their environment, which may shape self-supervised learning in important ways [100,104., 105., 106.]. Future work to measure and model this has the potential to improve understanding of infant learning and to guide new strategies for active learning in machines [107,108] (see Outstanding questions).

Given the importance of this early helpless period, what would happen if it was disrupted? In the domain of vision, a DNN model of disruption to early experience by cataracts suggests it changes representations in a way that impacts later function [83]. Early adversities, depending on their degree of impact, can either decelerate or accelerate early development [109]. To better understand the consequences of this on neurodevelopmental disorders or later cognitive functioning, longitudinal studies will be needed.

Outstanding questions Do brain systems that encode sensory inputs into rich embeddings develop during the helpless period while classification or policy systems do not?

Does the start of the development of symbolic representations and/or a language-of-thought mark the end of the helpless period?

Does self-supervised learning dominate in the helpless period and then transition to supervised learning from the end of the first year? This could be tested using neuroimaging (e.g., with fMRI or MEG) or behaviour (e.g., reaction times) to characterise the longitudinal development of representational geometry in the infant brain and comparing this to the changing representational geometry in self-supervised and supervised DNNs.

Does non-social learning proceed at a similar or greater rate to social learning during the helpless period?

What self-supervised learning objectives do human infants use? What inductive (learning) biases do human infants have due to their genetic programming and how does this improve their self-supervised learning?

To what extent is helplessness driven by motor limitations versus the need for self-supervised learning? Humans are bipedal, unlike arboreal primates, and there is evidence that such species differences in locomotor capabilities are linked to heterochronies during development. This raises the question of the extent to which helplessness is driven by motor limitations versus the need for self-supervised learning.

Why are humans more energy and sample efficient than machines and what are the implications of this difference in terms of their ability to learn new things?

Alt-text: Outstanding questions

## Glossary

**Adaptive behaviour**: behaviour that enables an animal to cope in their environment with greatest success.
**Altricial**: born immature and helpless.
**Behavioural extinction**: the cessation or loss of a behavioural pattern over time or generations due to environmental pressures.
**Deep neural network (DNN)**: an artificial neural network with many layers. An artificial neural network is a form of machine learning originally inspired and informed by knowledge about biological, typically human, brain organisation and processing.
**Embedding**: a relatively low-dimensional representation of a complex input.
**Encoder**: a block in a DNN that receives the input and derives an embedding that encodes the most relevant features.
**Event scale**: a cross-species average of the relative time of neurodevelopmental events.
**Foundation model**: a large DNN trained on a vast quantity of data, typically using self-supervised learning, that can act as the foundation for systems performing many different tasks.
**Helplessness**: a lack of adaptive behaviours that leads to reliance on caregivers.
**Heterochrony**: a relative acceleration of deceleration in the timing of specific biological processes.
**Precocial**: capable of movement and self-sufficiency soon after birth.
**Pre-training**: training an artificial neural network on one task or dataset, prior to training on the primary task or dataset of interest.
**Reinforcement learning**: learning actions that maximise a specified reward.
**Resting-state network**: a large-scale brain network comprising a set of regions that commonly co-activate, during rest or tasks.
**Self-supervised learning**: a machine learning strategy of identifying patterns within a data stream.
**Supervised learning**: training an artificial neural network with input examples that each have an explicit label.
